# *N*′-(4-Hy­droxy­benzyl­idene)thio­phene-2-carbohydrazide

**DOI:** 10.1107/S1600536810021483

**Published:** 2010-06-18

**Authors:** Yu-Feng Li, Jin-He Jiang, Fang-Fang Jian

**Affiliations:** a Microscale Science Institute, Department of Chemistry and Chemical Engineering, Weifang University, Weifang 261061, People’s Republic of China; b Microscale Science Institute, Weifang University, Weifang 261061, People’s Republic of China

## Abstract

In the title compound, C_12_H_10_N_2_O_2_S, the dihedral angle between the benzene and thio­phene rings is 23.34 (16)°. In the crystal structure, mol­ecules are linked by N—H⋯O and O—H⋯O hydrogen bonds, forming (100) sheets.

## Related literature

For background to the pharmacological properties of Schiff bases, see: Ren *et al.* (2002 ▶). For a related structure, see: Li *et al.* (2009 ▶).
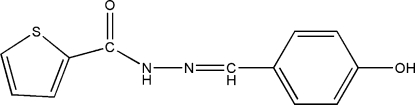


## Experimental

### 

#### Crystal data

C_12_H_10_N_2_O_2_S
*M* *_r_* = 246.28
Monoclinic, 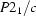

*a* = 9.5622 (19) Å
*b* = 12.404 (3) Å
*c* = 9.991 (2) Å
β = 104.40 (3)°
*V* = 1147.8 (4) Å^3^
*Z* = 4
Mo *K*α radiation
μ = 0.27 mm^−1^
*T* = 293 K
0.22 × 0.20 × 0.18 mm

#### Data collection

Bruker SMART CCD diffractometer
10889 measured reflections
2629 independent reflections
1501 reflections with *I* > 2σ(*I*)
*R*_int_ = 0.057

#### Refinement

*R*[*F* ^2^ > 2σ(*F* ^2^)] = 0.048
*wR*(*F* ^2^) = 0.181
*S* = 1.07
2629 reflections
154 parameters
H-atom parameters constrained
Δρ_max_ = 0.27 e Å^−3^
Δρ_min_ = −0.38 e Å^−3^

### 

Data collection: *SMART* (Bruker, 1997 ▶); cell refinement: *SAINT* (Bruker, 1997 ▶); data reduction: *SAINT*; program(s) used to solve structure: *SHELXS97* (Sheldrick, 2008 ▶); program(s) used to refine structure: *SHELXL97* (Sheldrick, 2008 ▶); molecular graphics: *SHELXTL* (Sheldrick, 2008 ▶); software used to prepare material for publication: *SHELXTL*.

## Supplementary Material

Crystal structure: contains datablocks global, I. DOI: 10.1107/S1600536810021483/hb5483sup1.cif

Structure factors: contains datablocks I. DOI: 10.1107/S1600536810021483/hb5483Isup2.hkl

Additional supplementary materials:  crystallographic information; 3D view; checkCIF report

## Figures and Tables

**Table 1:** Hydrogen-bond geometry (Å, °)

*D*—H⋯*A*	*D*—H	H⋯*A*	*D*⋯*A*	*D*—H⋯*A*
N1—H1*A*⋯O1^i^	0.86	2.09	2.887 (3)	154
O2—H2*C*⋯O1^ii^	0.82	2.10	2.913 (3)	174

Symmetry codes: (i) 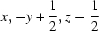; (ii) 
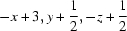
.

**Fig. 1.**
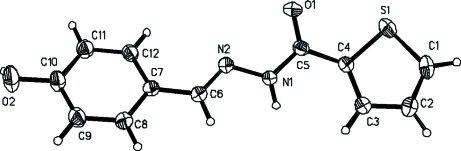
The structure of (I) showing 30% probability displacement ellipsoids.

## supplementary crystallographic information

### Comment

Schiff bases derivatives have attracted much attention due to their pharmacological activity (Ren *et al.*, 2002). As part of an investigation of the properties of Schiff bases functioning as ligands, we synthesized the title compound (I), and describe its structure here. The title compound contains two independent molecules in the unit. The dihedral angle between the aromatic rings is [23.33 (16)°]. In the crystal lattice, the N—H···O and O—H···O intramolecular hydrogen bonds which form the molecule structures.

Bond lengths and angles are comparable to those in a related compound (Li *et al.*, 2009).

### Experimental

A mixture of 4-methylbenzaldehyde (0.1 mol), and thiophene-2-carbohydrazide (0.1 mol) was stirred in refluxing ethanol (20 ml) for 4 h to afford the title compound (0.092 mol, yield 92%). Colourless blocks of (I) were obtained by recrystallization from ethanol at room temperature.

### Refinement

H atoms were fixed geometrically and allowed to ride on their attached atoms, with C—H distances=0.97 Å, and with *U*_iso_=1.2–1.5*U*_eq_.

### Crystal data

**Table:**

C_12_H_10_N_2_O_2_S	*F*(000) = 512
*M**_r_* = 246.28	*D*_x_ = 1.425 Mg m^−^^3^
Monoclinic, *P*2_1_/*c*	Mo *K*α radiation, λ = 0.71073 Å
Hall symbol: -P 2ybc	Cell parameters from 1501 reflections
*a* = 9.5622 (19) Å	θ = 3.1–27.5°
*b* = 12.404 (3) Å	µ = 0.27 mm^−^^1^
*c* = 9.991 (2) Å	*T* = 293 K
β = 104.40 (3)°	Block, colorless
*V* = 1147.8 (4) Å^3^	0.22 × 0.20 × 0.18 mm
*Z* = 4	

### Data collection

**Table:**

Bruker SMART CCD diffractometer	1501 reflections with *I* > 2σ(*I*)
Radiation source: fine-focus sealed tube	*R*_int_ = 0.057
graphite	θ_max_ = 27.5°, θ_min_ = 3.1°
phi and ω scans	*h* = −12→12
10889 measured reflections	*k* = −16→16
2629 independent reflections	*l* = −11→12

### Refinement

**Table:**

Refinement on *F*^2^	Primary atom site location: structure-invariant direct methods
Least-squares matrix: full	Secondary atom site location: difference Fourier map
*R*[*F*^2^ > 2σ(*F*^2^)] = 0.048	Hydrogen site location: inferred from neighbouring sites
*wR*(*F*^2^) = 0.181	H-atom parameters constrained
*S* = 1.07	*w* = 1/[σ^2^(*F*_o_^2^) + (0.1*P*)^2^] where *P* = (*F*_o_^2^ + 2*F*_c_^2^)/3
2629 reflections	(Δ/σ)_max_ < 0.001
154 parameters	Δρ_max_ = 0.27 e Å^−^^3^
0 restraints	Δρ_min_ = −0.37 e Å^−^^3^

### Special details

**Table:**

Geometry. All e.s.d.'s (except the e.s.d. in the dihedral angle between two l.s. planes) are estimated using the full covariance matrix. The cell e.s.d.'s are taken into account individually in the estimation of e.s.d.'s in distances, angles and torsion angles; correlations between e.s.d.'s in cell parameters are only used when they are defined by crystal symmetry. An approximate (isotropic) treatment of cell e.s.d.'s is used for estimating e.s.d.'s involving l.s. planes.
Refinement. Refinement of *F*^2^ against ALL reflections. The weighted *R*-factor *wR* and goodness of fit *S* are based on *F*^2^, conventional *R*-factors *R* are based on *F*, with *F* set to zero for negative *F*^2^. The threshold expression of *F*^2^ > σ(*F*^2^) is used only for calculating *R*-factors(gt) *etc*. and is not relevant to the choice of reflections for refinement. *R*-factors based on *F*^2^ are statistically about twice as large as those based on *F*, and *R*- factors based on ALL data will be even larger.

### Fractional atomic coordinates and isotropic or equivalent isotropic displacement parameters (Å^2^)

**Table:**

	*x*	*y*	*z*	*U*_iso_*/*U*_eq_	
S1	0.89443 (10)	0.07271 (7)	0.19012 (8)	0.0713 (3)	
N2	1.2232 (2)	0.37439 (19)	0.1118 (2)	0.0452 (6)	
O1	1.1172 (2)	0.24125 (17)	0.27388 (17)	0.0548 (6)	
N1	1.1217 (2)	0.29541 (19)	0.0602 (2)	0.0482 (6)	
H1A	1.0911	0.2863	−0.0275	0.058*	
C12	1.4377 (3)	0.5507 (2)	0.1730 (3)	0.0464 (6)	
H12A	1.4518	0.4976	0.2407	0.056*	
C4	0.9590 (3)	0.1566 (2)	0.0840 (2)	0.0435 (6)	
C5	1.0717 (3)	0.2334 (2)	0.1475 (2)	0.0406 (6)	
C7	1.3299 (3)	0.5378 (2)	0.0519 (2)	0.0430 (6)	
C6	1.2339 (3)	0.4464 (2)	0.0230 (3)	0.0483 (7)	
H6A	1.1759	0.4392	−0.0663	0.058*	
C11	1.5235 (3)	0.6408 (2)	0.1941 (3)	0.0506 (7)	
H11A	1.5936	0.6491	0.2767	0.061*	
O2	1.5867 (3)	0.81085 (19)	0.1088 (2)	0.0816 (8)	
H2C	1.6718	0.7957	0.1418	0.122*	
C10	1.5063 (3)	0.7198 (2)	0.0930 (3)	0.0518 (7)	
C8	1.3140 (3)	0.6189 (2)	−0.0471 (3)	0.0510 (7)	
H8A	1.2427	0.6120	−0.1291	0.061*	
C9	1.3999 (3)	0.7083 (2)	−0.0273 (3)	0.0569 (8)	
H9A	1.3866	0.7613	−0.0951	0.068*	
C3	0.8883 (4)	0.1398 (3)	−0.0502 (3)	0.0647 (9)	
H3A	0.9077	0.1779	−0.1237	0.078*	
C2	0.7834 (4)	0.0591 (3)	−0.0662 (3)	0.0756 (11)	
H2B	0.7253	0.0377	−0.1512	0.091*	
C1	0.7759 (4)	0.0163 (3)	0.0548 (3)	0.0765 (11)	
H1B	0.7124	−0.0386	0.0632	0.092*	

### Atomic displacement parameters (Å^2^)

**Table:**

	*U*^11^	*U*^22^	*U*^33^	*U*^12^	*U*^13^	*U*^23^
S1	0.0791 (6)	0.0780 (6)	0.0592 (5)	−0.0243 (5)	0.0220 (4)	0.0166 (4)
N2	0.0506 (13)	0.0473 (14)	0.0398 (10)	−0.0127 (10)	0.0151 (10)	−0.0051 (9)
O1	0.0635 (13)	0.0682 (14)	0.0331 (9)	−0.0089 (10)	0.0129 (8)	0.0017 (8)
N1	0.0592 (15)	0.0545 (14)	0.0323 (10)	−0.0207 (11)	0.0138 (9)	−0.0059 (9)
C12	0.0476 (16)	0.0448 (15)	0.0466 (13)	−0.0047 (12)	0.0113 (12)	0.0066 (11)
C4	0.0461 (15)	0.0454 (15)	0.0416 (13)	−0.0069 (12)	0.0155 (11)	0.0010 (10)
C5	0.0446 (15)	0.0421 (14)	0.0374 (12)	−0.0003 (11)	0.0145 (10)	0.0003 (10)
C7	0.0485 (15)	0.0430 (15)	0.0398 (12)	−0.0050 (12)	0.0151 (11)	−0.0050 (10)
C6	0.0552 (17)	0.0510 (16)	0.0393 (13)	−0.0130 (13)	0.0129 (12)	−0.0049 (11)
C11	0.0465 (16)	0.0493 (17)	0.0514 (14)	−0.0048 (13)	0.0035 (12)	0.0062 (12)
O2	0.0694 (16)	0.0573 (15)	0.0975 (17)	−0.0248 (12)	−0.0184 (13)	0.0290 (12)
C10	0.0473 (16)	0.0409 (16)	0.0640 (17)	−0.0058 (13)	0.0078 (13)	0.0081 (12)
C8	0.0577 (18)	0.0497 (17)	0.0423 (13)	−0.0090 (14)	0.0064 (12)	0.0018 (11)
C9	0.063 (2)	0.0483 (18)	0.0543 (15)	−0.0072 (14)	0.0048 (14)	0.0120 (12)
C3	0.075 (2)	0.074 (2)	0.0457 (15)	−0.0327 (18)	0.0160 (15)	−0.0009 (14)
C2	0.079 (2)	0.085 (3)	0.0599 (18)	−0.041 (2)	0.0132 (17)	−0.0104 (16)
C1	0.073 (2)	0.071 (2)	0.088 (2)	−0.0361 (19)	0.0245 (19)	0.0017 (18)

### Geometric parameters (Å, °)

**Table:**

S1—C1	1.685 (4)	C6—H6A	0.9300
S1—C4	1.707 (2)	C11—C10	1.387 (4)
N2—C6	1.281 (3)	C11—H11A	0.9300
N2—N1	1.385 (3)	O2—C10	1.354 (3)
O1—C5	1.233 (3)	O2—H2C	0.8200
N1—C5	1.337 (3)	C10—C9	1.375 (4)
N1—H1A	0.8600	C8—C9	1.364 (4)
C12—C11	1.371 (4)	C8—H8A	0.9300
C12—C7	1.390 (4)	C9—H9A	0.9300
C12—H12A	0.9300	C3—C2	1.398 (4)
C4—C3	1.360 (4)	C3—H3A	0.9300
C4—C5	1.461 (4)	C2—C1	1.339 (4)
C7—C8	1.392 (4)	C2—H2B	0.9300
C7—C6	1.443 (4)	C1—H1B	0.9300
			
C1—S1—C4	91.70 (14)	C12—C11—H11A	119.8
C6—N2—N1	113.9 (2)	C10—C11—H11A	119.8
C5—N1—N2	119.68 (19)	C10—O2—H2C	109.5
C5—N1—H1A	120.2	O2—C10—C9	117.6 (3)
N2—N1—H1A	120.2	O2—C10—C11	122.9 (3)
C11—C12—C7	120.9 (2)	C9—C10—C11	119.5 (3)
C11—C12—H12A	119.6	C9—C8—C7	121.9 (3)
C7—C12—H12A	119.6	C9—C8—H8A	119.1
C3—C4—C5	131.4 (2)	C7—C8—H8A	119.1
C3—C4—S1	110.6 (2)	C8—C9—C10	119.9 (3)
C5—C4—S1	118.01 (18)	C8—C9—H9A	120.0
O1—C5—N1	122.0 (2)	C10—C9—H9A	120.0
O1—C5—C4	122.1 (2)	C4—C3—C2	112.9 (3)
N1—C5—C4	115.9 (2)	C4—C3—H3A	123.5
C12—C7—C8	117.5 (3)	C2—C3—H3A	123.5
C12—C7—C6	124.2 (2)	C1—C2—C3	112.3 (3)
C8—C7—C6	118.3 (2)	C1—C2—H2B	123.9
N2—C6—C7	124.5 (2)	C3—C2—H2B	123.9
N2—C6—H6A	117.8	C2—C1—S1	112.6 (3)
C7—C6—H6A	117.8	C2—C1—H1B	123.7
C12—C11—C10	120.3 (3)	S1—C1—H1B	123.7

### Hydrogen-bond geometry (Å, °)

**Table:**

*D*—H···*A*	*D*—H	H···*A*	*D*···*A*	*D*—H···*A*
N1—H1A···O1^i^	0.86	2.09	2.887 (3)	154
O2—H2C···O1^ii^	0.82	2.10	2.913 (3)	174

Symmetry codes: (i) *x*, −*y*+1/2, *z*−1/2; (ii) −*x*+3, *y*+1/2, −*z*+1/2.
